# Etoposide promotes DNA loop trapping and barrier formation by topoisomerase II

**DOI:** 10.1038/s41589-022-01235-9

**Published:** 2023-01-30

**Authors:** Tung T. Le, Meiling Wu, Joyce H. Lee, Neti Bhatt, James T. Inman, James M. Berger, Michelle D. Wang

**Affiliations:** 1 Howard Hughes Medical Institute, Cornell University, Ithaca, NY USA; 2 Department of Physics and LASSP, Cornell University, Ithaca, NY USA; 3 Department of Biophysics and Biophysical Chemistry, Johns Hopkins University School of Medicine, Baltimore, MD USA

**Keywords:** Single-molecule biophysics, DNA damage and repair, DNA

## Abstract

Etoposide is a broadly employed chemotherapeutic and eukaryotic topoisomerase II poison that stabilizes cleaved DNA intermediates to promote DNA breakage and cytotoxicity. How etoposide perturbs topoisomerase dynamics is not known. Here we investigated the action of etoposide on yeast topoisomerase II, human topoisomerase IIα and human topoisomerase IIβ using several sensitive single-molecule detection methods. Unexpectedly, we found that etoposide induces topoisomerase to trap DNA loops, compacting DNA and restructuring DNA topology. Loop trapping occurs after ATP hydrolysis but before strand ejection from the enzyme. Although etoposide decreases the innate stability of topoisomerase dimers, it increases the ability of the enzyme to act as a stable roadblock. Interestingly, the three topoisomerases show similar etoposide-mediated resistance to dimer separation and sliding along DNA but different abilities to compact DNA and chirally relax DNA supercoils. These data provide unique mechanistic insights into the functional consequences of etoposide on topoisomerase II dynamics.

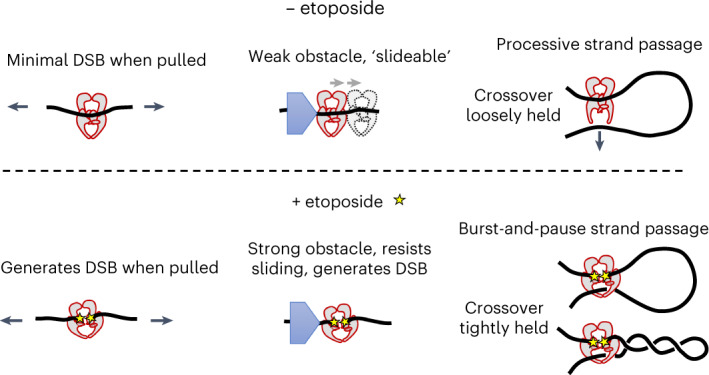

## Abstract (web summary)

Using single-molecule biophysics methods, Le et al. discovered that etoposide, a chemotherapeutic poison of topoisomerase II (topo II), promotes topo II to compact DNA, trap DNA loops and pause DNA supercoiling relaxation, thus converting topo II into a strong roadblock to DNA processing.

## Main

The double-helical structure of genomic DNA results in supercoiling and topological entanglements during fundamental processes, such as transcription and DNA replication. The excessive torsional buildup, as well as DNA catenanes and knots, can impede these processes, and topoisomerases are essential for resolving such topological challenges.

Type IIA topoisomerases (topo IIs) constitute one topoisomerase family essential for cell viability. Most eukaryotes, such as *Saccharomyces cerevisiae*, express a single topo II isoform, whereas vertebrates have two: topo IIα and topo IIβ^1^. Yeast topo II is indispensable in chromosome segregation^2^ and facilitates the transcription of long genes^3^. Human topo II isoforms are more specialized in cellular functionality, with topo IIα participating primarily in DNA replication and chromosome segregation and topo IIβ playing a crucial role in transcription regulation^1^.

**Fig. 1:**
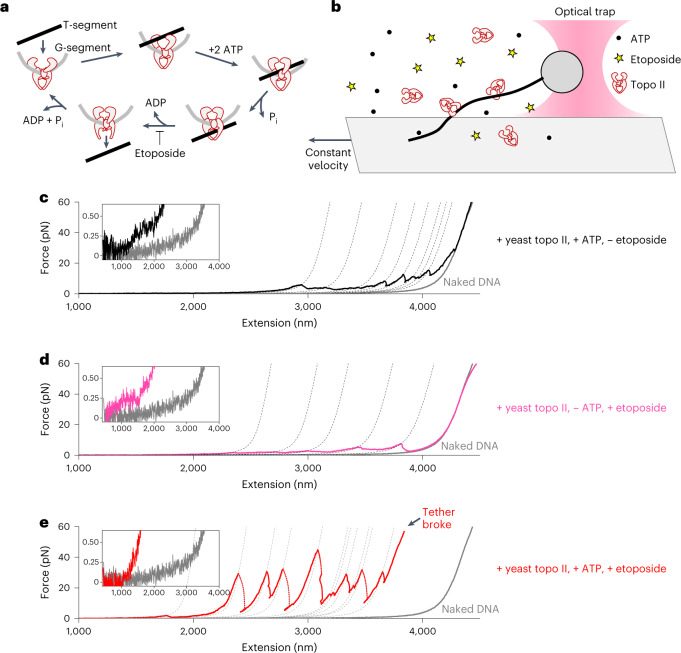
Direct measurement of etoposide-mediated DNA loop formation and DNA break stability in topo II by stretching DNA at a constant velocity. **a**, Cartoon showing the kinetic steps of topo II catalytic cycle. **b**, Experimental configuration for stretching DNA under a constant velocity. A 12.7-kb dsDNA was anchored between a coverslip surface and a streptavidin-coated polystyrene bead. After topo II and ATP were introduced into the sample chamber with or without etoposide, the bead was captured by an optical trap, and the DNA tether was stretched as the coverslip moved at a constant velocity of 200 nm s^−1^. The bead position was clamped by modulating laser power. **c**, A representative force versus extension curve in the presence of 5 pM yeast topo II, 1 mM ATP and no etoposide (black curve). The dashed gray lines indicate the worm-like chain curves that pass through the detected force peaks. For comparison, a typical curve of the naked DNA is also shown. The inset shows a region near 0.5 pN during the initial low-force stretch. **d**, Same as **c** except without ATP but with 100 µM etoposide (pink). **e**, Same as **c** except that with 100 µM etoposide (red). The black arrow indicates the point of detected tether breakage. Source data

All eukaryotic topo IIs are homodimers and share significant homology in sequence and structure^4–6^. These enzymes also share a similar ATP-dependent strand passage mechanism^1^ (Fig. 1a). During a catalytic cycle, topo II binds to a primary segment of DNA (the gate-segment, or G-segment) and then captures a second DNA duplex (the transport-segment, or T-segment). Upon binding to ATP, one dimer interface (termed the N-gate) closes around the T-segment, leading topo II to create a transient break in the G-segment DNA by the formation of a covalent enzyme–DNA link. Next, the region binding the cleaved G-segment (the DNA-gate) separates, allowing the T-segment to pass through the transiently opened DNA-gate. After passage, the G-segment is resealed, and the T-segment is expelled by the temporary opening of a C-terminal dimerization interface (the C-gate), allowing the enzyme to restart its catalytic cycle^7,8^.

In a typical catalytic cycle, the DNA break formed by topo II is transient. However, if topo II fails to religate this cleaved intermediate, permanent double-stranded breaks (DSBs) can accumulate, threaten genome stability and ultimately induce cell death^9,10^. This vulnerability has been exploited in developing effective small molecule therapeutics that target topo II to kill proliferating cancerous cells. A potent group of anti-cancer drugs, commonly referred to as ‘topoisomerase poisons’, directly prevents DNA religation by stabilizing the cleavage complex^11,12^. If the cleaved complex persists, it can be converted into a DSB, eliciting cell death^13,14^.

Etoposide is a topo II poison with broad clinical use as a chemotherapeutic agent^15,16^. Although a molecular picture of how etoposide binds to the topo II–DNA cleavage complex to stabilize break formation is known^6,12^, whether and how etoposide can also interfere with enzyme dynamics has remained unclear. It has been hypothesized that an etoposide-mediated DNA cleavage complex presents a physical obstacle against the progression of other DNA-based processes, such as transcription and DNA replication, and attempts to overcome such barriers result in DNA breaks^13^. However, direct physical evidence that the cleavage complex represents a highly stable barrier has been lacking.

In the present work, we investigated the physical and dynamic impact of etoposide on yeast topo II, human topo IIα and human topo IIβ. We examined the mobility of bound topo II sliding on DNA, the force required to disrupt topo II binding at a DNA crossover and the force required to expose the DNA break in a cleavage complex. We also investigated how etoposide alters these physical properties and the relaxation of DNA supercoiling by topo II.

## Results

### Direct detection of topo II-induced DNA loop formation

**Extended Data Fig. 1:**
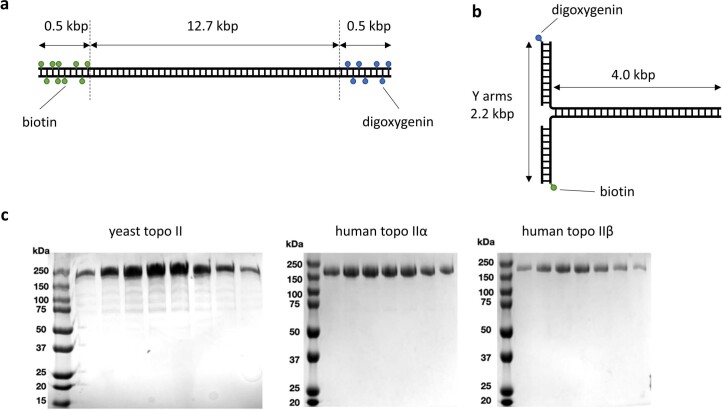
DNA templates and protein gels. (a) DNA stretching template. The DNA stretching template contained a 12.7 kb dsDNA, flanked by two ~ 500 bp DNA adaptors, which were multi-labeled with biotin at one end and digoxygenin at the other end. For more details, see Methods. (b) DNA unzipping template. The DNA unzipping template consisted of a 4.0 kb DNA unzipping segment, with its two strands connected to two dsDNA adaptor arms. The end of one arm was labeled with a single biotin tag, and the end of the other arm was labeled with a single digoxygenin tag. (c) SDS-PAGE analysis of peak, purified topo II fractions of the three topo II isoforms obtained from gel filtration chromatography. A molecular weight ladder is shown at the left of each gel. Yeast topo II, human topo IIα, and human topo IIβ have an expected molecular weight of 164 kDa, 174 kDa, and 183 kDa, respectively. Yeast topo II occasionally can be seen to migrate with a somewhat larger than expected molecular weight depending on the gradient gel composition used and running conditions. Each experiment was conducted at least 3 times. Source data

To investigate the strength of the interaction between topo II and DNA, we employed a single-molecule DNA stretching assay using an optical trap (also referred to as optical tweezers) to examine budding yeast topo II, human topo IIα and human topo IIβ (Extended Data Fig. 1). In a stretching assay, a double-stranded DNA (dsDNA) molecule (Fig. 1b and Extended Data Fig. 1a) was anchored between the surface of a sample chamber microscope coverslip and an optically trapped microsphere. The DNA was then stretched as the coverslip was moved away from the optical trap at a constant velocity^17^ (Methods).

In the presence of topo II (Fig. 1c), as a DNA molecule was stretched, the force began to rise above the naked DNA force baseline^18,19^, suggesting DNA tether shortening due to topo II interactions with the DNA. Previous studies using magnetic tweezers (MT) also reported that *Drosophila* topo II and topo IV shorten DNA extension^20,21^.

**Extended Data Fig. 2:**
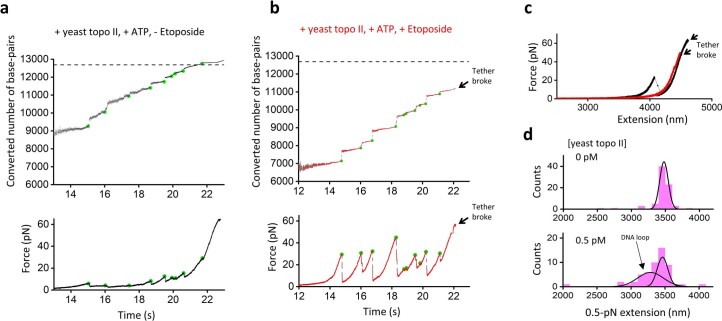
DNA loop size determination and DNA loop capture at a very low topo II concentration. (a) The number of base pairs (upper panel) and force (lower panel) as a function of time for data shown in Fig. 1c. Number of DNA base pairs was converted from the force and extension of the DNA. For this conversion, we used the modified Marko-Siggia formula^19^ with a 45 nm persistence length and a 1200 pN stretching modulus. Green circles indicate the locations of the detected disruption force peaks (see Methods). The length change (in bp) between two adjacent green circles provides the DNA loop size. The horizontal dashed line indicates the full length of the naked DNA. (b) The number of base pairs (upper panel) and force (lower panel) as a function of time for data shown in Fig. 1e. The conversion method was the same as for (a). (c) Constant-velocity stretching curves of DNA tethers in a condition with a very low yeast topo II concentration of 0.5 pM, 1 mM ATP, and 100 µM etoposide. Two typical force-extension traces are shown: one without any DNA loop disruption (red) and another with a single DNA loop disruption (black). (d) DNA extension at 0.5 pN in the presence or absence of 0.5 pM yeast topo II, with 100 µM etoposide and 1 mM ATP (*N* = 60 biologically independent traces at each condition). In the absence of topo II, the extension histogram showed a single Gaussian distribution, while in the presence of topo II, an additional population with a shorter extension was detected, corresponding to tethers with DNA loops with an average size of ~ 500 bp. Source data

As stretching continued, clear force peaks were detected in a topo II concentration-dependent manner. At each force peak, the force rise was followed by a sudden force drop with a concurrent increase in DNA length, indicating disruption of topo II–DNA interactions and subsequent release of DNA captured by topo II. Force peaks recurred throughout stretching until the DNA reached its full extension. When etoposide was introduced with topo II but without ATP, these force features did not change substantially (Fig. 1d). Interestingly, when ATP was also present, the force peaks became much more pronounced, and the DNA tether broke more frequently during stretching (Fig. 1e and Extended Data Fig. 2a,b). These behaviors demonstrate that the action of etoposide is linked to the ATPase cycle of topo II.

**Fig. 2:**
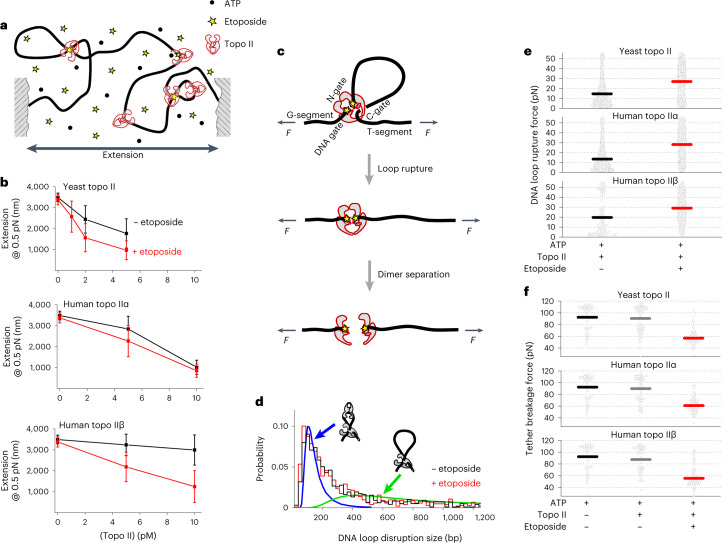
Etoposide enhances topo II binding at a DNA crossover and reduces topo II dimer separation force. **a**, Cartoon illustrating topo II compaction of DNA. **b**, DNA extension measured at 0.5 pN is plotted against topo II concentration in the presence of 1 mM ATP and 100 µM etoposide. Shown are the mean values and their standard deviations obtained from ~140 biologically independent traces for each condition. The same traces and statistics were also used for data analysis of **d**, **e** and **f**. **c**, Proposed model for topo II-mediated DNA loop formation and DSB in the presence of etoposide and ATP. When topo II captures a DNA crossing, the G-segment forms a cleavage complex, whereas the T-segment becomes stably trapped by topo II (depicted here between the DNA-gate and a closed C-gate of the enzyme), securing the DNA crossing. Pulling on the two DNA ends promotes DNA loop rupture by yanking the T-strand out of the C-gate (step 1). Further pulling will lead to topo II dimer separation (step 2) and DSB formation. **d**, Histograms of the DNA loop size in the presence of 100 µM etoposide with 5 pM yeast topo II and 1 mM ATP. The loop size of each disruption event (Extended Data Fig. 2a,b) was pooled to obtain each histogram. Also shown are numerical predictions by considering a combination of two possibilities: DNA loop formation without any intrinsic bend (green) or with a sharp DNA bend of 150° introduced by another bound topo II (blue). The looping probability or *J*-factor of non-bent or bent DNA (Extended Data Fig. 3) was scaled to match the DNA loop size histogram. **e**, DNA loop rupture force. Data were taken under conditions with 100 µM etoposide, 1 mM ATP and 5 pM yeast topo II, 10 pM human topo IIα or 10 pM human topo IIβ. Each dot plot shows the different loop rupture force events, with their mean force shown (solid horizontal line). **f**, DNA tether breakage force. Each dot plot shows different tether breakage force events, with their mean force shown (solid horizontal line). Source data

An initial shortening of the DNA tether in the stretching traces suggested that topo II induces DNA compaction (Fig. 1c–e, insets, and Methods). For all three topo II isoforms, we found that DNA extension (measured at 0.5 pN) decreased as topo II concentration increased (Fig. 2a,b). Based on the topo II concentration required to compact the DNA to the same extent, yeast topo II compacted DNA most effectively, whereas human topo IIβ compacted DNA least effectively. The presence of etoposide enhanced DNA compaction, particularly for yeast topo II and human topo IIβ.

Each force peak in a force–extension curve corresponds to a sudden release of DNA initially captured by topo II (step 1 of Fig. 2c). Topo II could capture DNA via a DNA loop, likely by binding to a DNA crossover formed from both the G-segment and the T-segment^22,23^, and the applied tension in the DNA may disrupt topo II binding at this crossover, releasing the DNA captured in the loop. Because topo II-mediated DNA loop formation was detected even at a very low topo II concentration (Extended Data Fig. 2c,d), it is possible that a single topo II can capture such a loop.

**Extended Data Fig. 3:**
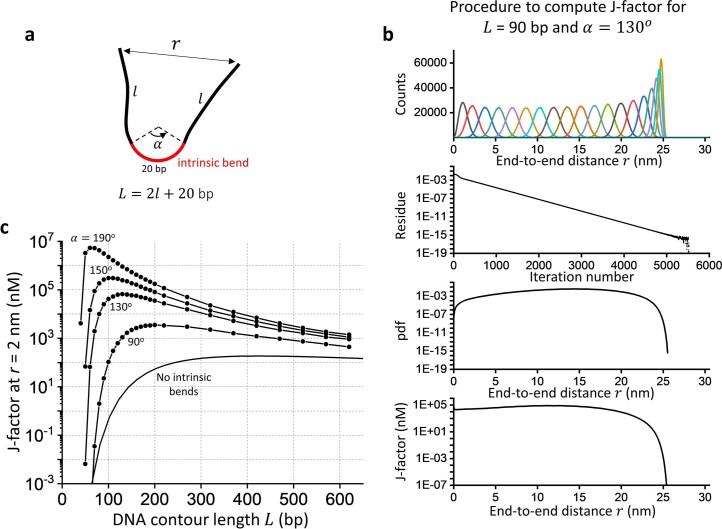
Monte-Carlo (MC) simulations to obtain the looping probability of a DNA with an intrinsic bend. (a) DNA is modeled as a chain of discretized segments of 5 bp in length, with a total contour length of *L*=2*l*+20 bp. The intrinsic bend (red, 20 bp) is modeled as an arc, mimicking the footprint of a bound topo II on DNA. This bend portion is assumed to be rigid and constrained by topo II-DNA interactions, while the remaining DNA (2*l*) behaves like a worm-like chain with a persistence length of 45 nm. The angle *α* indicates the bending imposed by the DNA-bound topo II, which is ~ 130 – 150^o^ from previous structural studies^5,59^. (b) Procedure to calculate DNA-looping probability (or *J*-factor) illustrated using *L*= 90 bp and *α* = 130^o^. From top to bottom: biased end-to-end distance distributions with varied end-to-end distance *r*_0_ from 0 to 90 bp in 5 bp increment in the biased potential $U = \frac{1}{2}k\left( {r - r_0} \right)^2$ with *k* = 0.8946 pN∙nm/bp^2^ were obtained from MC simulations with an umbrella sampling (see Methods). Each histogram was obtained with 1.5×10^6^ MC steps. To deduce the unbiased probability density function (pdf) of the end-to-end distance, we performed the Weighted Histogram Analysis Method (WHAM). This involves 1) solving for the pdf numerically through iteration until the solution converges, that is, the residue (the average difference of the pdf over consecutive iteration step) reaches zero, 2) reconstructing the unbiased pdf from biased pdfs using WHAM, and 3) calculating the looping probability *J*-factor from the unbiased pdf (see Methods). (c) The looping probability *J*-factor (in nM) calculated at *r* = 2*nm* for DNA with varied contour length and intrinsic bending angle *α*. The solid line shows the *J*-factor at *r* = 2*nm* for DNA without an intrinsic bend obtained by a semi-analytical calculation from Douarche and Cocco^55^. Source data

We characterized the amount of DNA release (referred to as DNA loop size) in each force disruption event (Extended Data Fig. 2a,b). The distribution of the loop size is broad, showing a peak at ~100 base pairs (bp) and a long tail in the distribution (Fig. 2d). Monte Carlo (MC) simulations (Methods and Extended Data Fig. 3) indicate that this distribution may be a combination of two populations: a long-loop population (several hundreds of base pairs in size) that can be described by a simple DNA loop formation model (green curve in Fig. 2d)^24,25^ and a short-loop population (~100 bp in size) that requires a sharp DNA bend within the DNA loop (blue curve in Fig. 2d).

**Extended Data Fig. 4:**
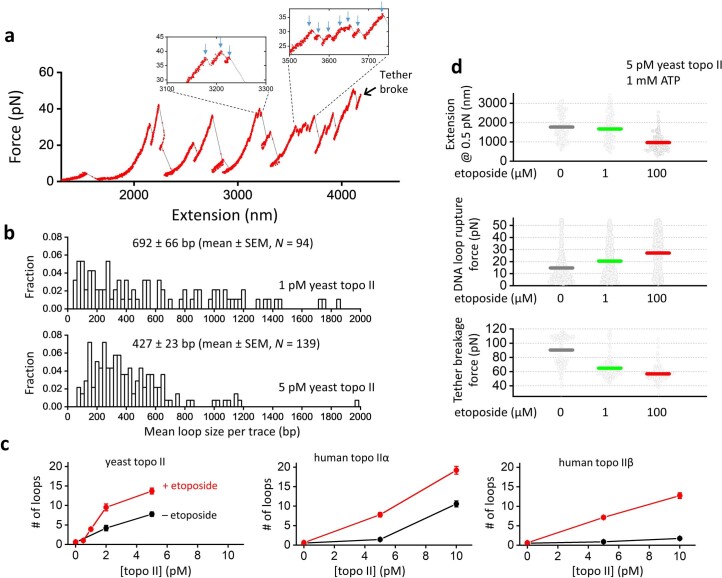
DNA looping and breakage characterization. (a) A typical trace under the constant-velocity stretching mode with 5 pM yeast topo II, 100 µM etoposide, and 1 mM ATP. Inset boxes show the zoom-in view of some disruption events (blue arrows). Note that small disruption events (vertical blue arrows) tend to cluster. (b) Histograms of the average DNA loop size of each stretching trace at different topo II concentrations with 100 µM etoposide and 1 mM ATP. DNA loop size per tether was obtained from the mean of all disruption events of that tether, and loop sizes from different tether were pooled for the histograms. (c) Number of loops per tether. To determine if the addition of etoposide increases the probability of topo II-mediated loop capture in the stretching experiments, we counted the loop disruption events to calculate the number of loops captured for each trace of the data used in Fig. 2e. If a trace broke before reaching its full contour length as could occur when etoposide was present, we scaled the number of loops from the direct counting by the ratio of the full DNA contour length to the DNA contour length at the breakage point. This treatment should give a reasonable estimate of all loops in each tether. This analysis shows that the presence of etoposide increases the number of loops captured for all three topo II isoforms. For each condition, data were pooled from *N* ~ 140 biologically independent traces. Error bars are SEMs. (d) DNA extension at 0.5 pN, loop rupture force, and tether breakage force as a function of etoposide concentration. For each condition, data were pooled from *N* ~ 150 biologically independent traces (dot plot), with the mean indicated as a solid horizontal line. Source data

The short-loop (<200 bp) population could arise from the binding of another topo II molecule within the loop or possible bending or wrapping activity manifested by topo II itself^26^. Alternatively, this population may also result from ‘stick-and-slip’ of topo II interactions with the T-strand as the size of a single loop is reduced under pulling, resulting in clustered, smaller DNA lengthening events in some traces (Extended Data Fig. 4a). The mean loop size for each trace reduces with higher enzyme concentrations (Extended Data Fig. 4b), consistent with multi-topo II binding contributing to the short-loop population.

The presence of etoposide with topo II and ATP did not substantially change the loop size distribution (Fig. 2d) but increased the number of loops trapped per trace for all three types of topo isoforms (Extended Data Fig. 4c). Notably, the presence of etoposide increased the loop rupture force (Figs. 1e and 2e and Extended Data Fig. 4d). The mean loop rupture force was about 16 pN for the three topo II isoforms in the absence of etoposide and increased to about 28 pN in the presence of etoposide and ATP, demonstrating that etoposide enhances topo II-mediated DNA loop stabilization.

### Direct detection of topo II-mediated DNA breaks

The hallmark of etoposide’s action on topo II is the stabilization of a cleavage complex^11,12^. Methods for detecting the cleavage complex typically rely on treating topo II–DNA reactions with strong detergents to denature the protein and expose the DSB^11,27^. With those approaches, capturing an accurate snapshot of the cleavage complex population is difficult without altering the dynamics of gate opening and closing of topo II^28^.

The stretching method described here is highly sensitive to DNA breaks and provides a direct detection approach. We observed that, in the absence of topo II, DNA tethers could withstand a very high force, with a mean breakage force of about 92 pN (Fig. 2f). The presence of topo II and ATP without etoposide slightly decreased the mean to about 89 pN. The addition of etoposide reduced this mean DNA breakage force for all three topo II isoforms to about 58 pN (Figs. 1e and 2f), consistent with etoposide increasing the frequency of topo II-mediated DNA tether breakage.

We interpret the increase seen for topo II-mediated tether breakage to result from enzyme dimer separation under tension applied to the DNA (step 2 in Fig. 2c). When topo II forms a cleavage complex, each topo II monomer becomes covalently linked to one end of a cleaved DNA segment^12,29,30^. As the two DNA segments are pulled away from each other under tension, the two monomers could separate, resulting in tether breakage.

**Fig. 3:**
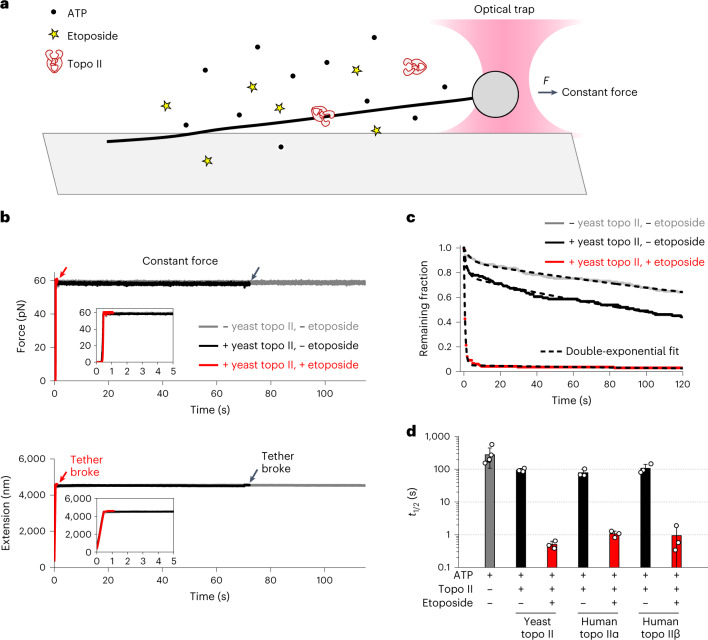
Direct measurement of etoposide-mediated DNA breakage of topo II by stretching DNA at a constant force. **a**, Experimental configuration for stretching DNA under a constant force. Topo II and ATP were introduced into the sample chamber with or without etoposide. A DNA tether was then rapidly stretched and subsequently held under a constant force of 60 pN using the force-clamp mode of an optical trap. In this mode, the laser power was held constant, and the bead displacement from the trap center was constantly maintained by modulating the coverslip position via a piezo stage. A tether breakage event was detected by a sudden and rapid movement of the piezo. These experiments included 1 pM yeast topo II, 3 pM human topo IIα or 3 pM human topo IIβ and were conducted with 1 mM ATP and with or without 100 µM etoposide. **b**, Representative traces of force versus time (top) and the corresponding extension versus time (bottom). After the initial force ramp to the force set point, the force in the DNA was held constant. Arrows indicate the timepoints of tether breakage. The insets show regions of first 5 seconds. **c**, Example of tether remaining probability as a function of time. Shown are data for naked DNA alone (gray), DNA with 1 pM yeast topo II and 1 mM ATP without etoposide (black) and DNA with 1 pM yeast topo II, 1 mM ATP and 100 µM etoposide (red). Each distribution is fitted with a double exponential function (dashed curves) to obtain the characteristic half-life (*t*_1/2_). For fit parameters of all three topo II isoforms, see Source Data file. **d**, Half-lives of DNA tether for the three topo II isoforms. Each half-life (mean ± s.d.) was obtained from *n* ≥ 3 biologically independent sample chambers, each with about 30–50 stretching traces. Source data

To more accurately investigate topo II-mediated DNA tether breakage, we used a constant-force stretching experiment where each DNA tether was rapidly stretched to 60 pN and then held at this force via the force-clamp mode of the optical trap (Fig. 3a,b and Methods). In this method, increased levels of tether breakage result in a net reduction in the lifetime of the DNA tether withstanding this force. For these experiments, we used a very low topo II concentration to reduce the frequency of DNA loop formation (Extended Data Fig. 2c,d). We then plotted the fraction of the remaining tethers as a function of time by pooling measurements from multiple DNA tethers. Each plot was fit by a double exponential function (Fig. 3c). For simplicity, we used the half-life from the fit (*t*_1/2_) to characterize the tether lifetime.

As shown in Fig. 3d, for DNA alone, the tether half-life was 270 seconds. The tether lifetime under this condition was likely limited by DNA end anchor detachment from the anti-digoxigenin-coated surface or the streptavidin-coated bead. The presence of topo II decreased the tether half-life to about 100 seconds for all three topo II isoforms tested, likely due to transient topo II-mediated DNA breaks that occurred during a normal catalytic cycle, demonstrating an exceptional level of sensitivity for the method.

**Extended Data Fig. 5:**
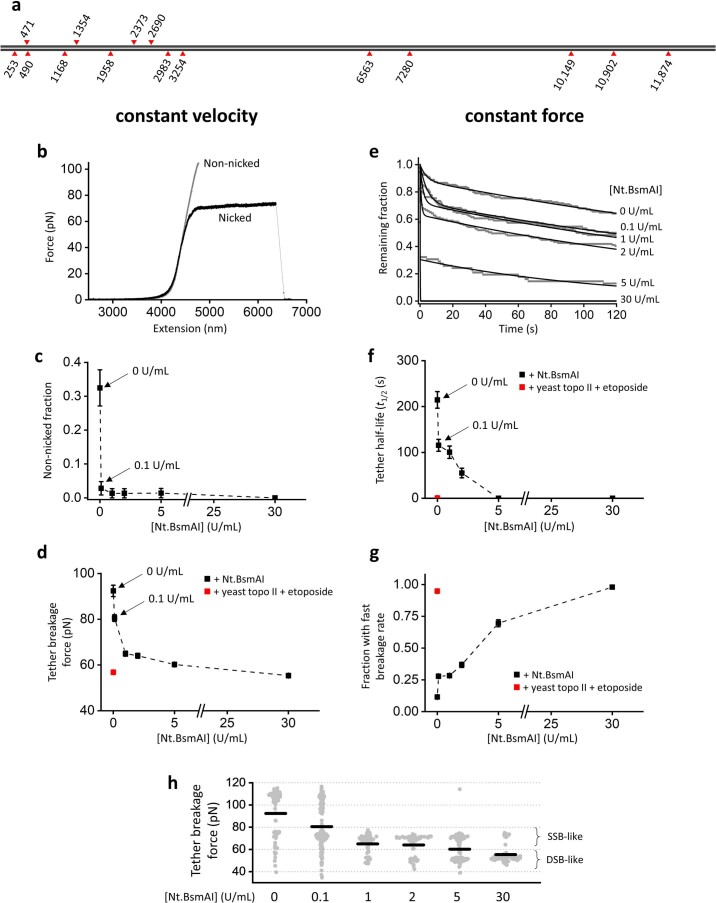
Stretching of a nicked DNA tether. (a) Location of the nicking sites of Nt.BsmAI on the 12.7 kb DNA stretching template. Upper triangles indicate nicking sites on the top strand, while lower triangles indicate nicking sites on the bottom strand. (b) Representative stretching curves of a DNA tether without nicks and with at least one nick. A nicked DNA molecule shows the over-stretching transition around 65 pN^60^, while an un-nicked torsionally constrained DNA molecule does not show this transition. (c) Nicked fraction as a function of nickase concentration with 15 min reaction. For each nickase concentration, data were pooled from *N* ≥ 100 biologically independent traces. Error bars are s.e.m. values. The same traces and statistics were also used for analysis in (d) and (h). (d) Mean tether breakage force (and s.e.m.) under constant-velocity stretching. The data point for yeast topo II with etoposide (Fig. 2f) is shown for comparison. (e) Tether remaining fraction versus time during the constant-force (60 pN) stretching. Each data curve was (grey) fitted with a double-exponential function (black curve). (f) DNA tether characteristic lifetime *t*_1/2_. For each condition, *N* ≥ 60 biologically independent traces. Error bars are uncertainties of the fit parameters. The same traces and statistics were also used for the analysis in (g). (g) Fraction of tethers with a faster breakage rate, obtained from the double-exponential fit to the survival fraction in (e). Error bars are uncertainties of the fit parameters. (h) Tether-breakage force versus nickase concentration for the same data as those for (d) except in the dot plot format. We interpret the population centered around 71 pN as single-stranded breaks and the population centered around 50 pN as double-stranded breaks, which could occur when two nicks are in close proximity. Source data

When etoposide was also present, the tether half-life was reduced more than 100-fold (to ≤1.0 seconds) compared to that of either the naked DNA or topo II. Control experiments using a DNA nicking enzyme suggest that DNA breakage observed with topo II in the presence of etoposide derives from dsDNA breaks and not single-stranded DNA (ssDNA) nicks (Extended Data Fig. 5). A control experiment using a lower concentration of etoposide, which favors ssDNA breaks^31^, showed much-reduced DNA loop stabilization (Extended Data Fig. 4d), indicating that full DNA breakage is coupled with loop trapping.

Altogether, the approaches developed here provide the first direct measurement of the overall stability of the topo II dimer in the absence or presence of etoposide by mechanical separation. Our data from both the constant-velocity stretching and constant-force stretching suggest that etoposide binding destabilizes the DNA–topo II complex overall.

### ATP hydrolysis is required for etoposide-induced dynamics

The topo II catalytic reaction requires chemomechanical coordination between different domains of the enzyme and its ATP hydrolysis cycle. Previous studies found that ATP binding allows topo II to capture a T-segment, whereas ATP hydrolysis (at least for yeast topo II) biases DNA-gate opening for the subsequent transfer of the T-segment through the DNA-gate^5,22^. To further examine the role of ATP on the action of etoposide, we systematically examined topo II-mediated loop capture and topo II-mediated DNA breaks in the absence of either ATP binding or ATP hydrolysis.

**Fig. 4:**
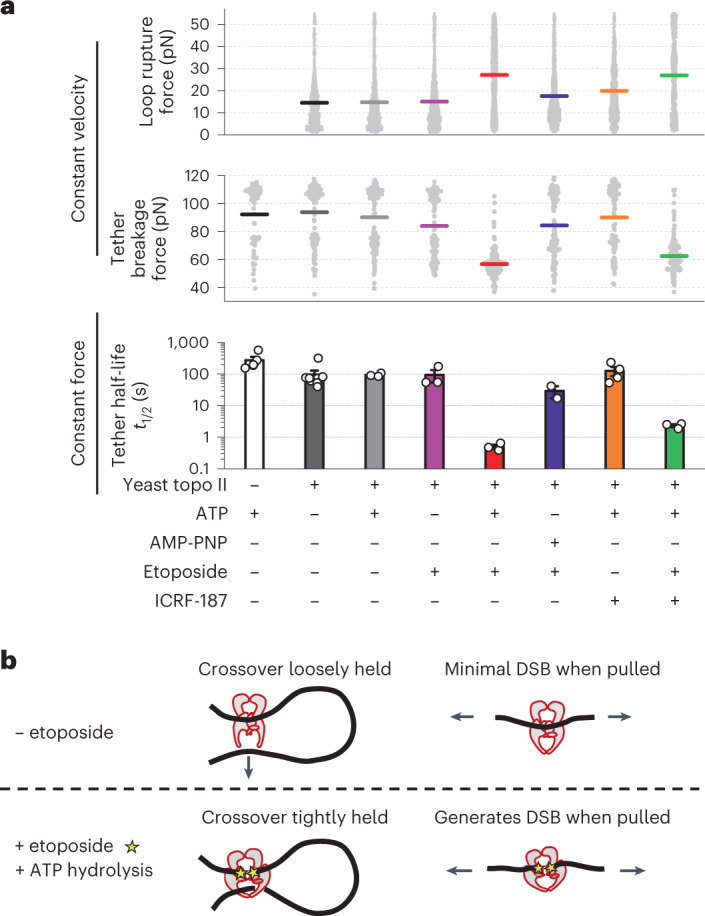
ATP hydrolysis is required for etoposide-enhanced DNA crossover capture and DSB generation. **a**, The effect of etoposide on DNA crossover capture and DSB generation. The top two panels show the loop rupture force and the tether breakage force from the constant-velocity stretching experiments. Each dot plot shows different loop rupture events or tether breakage events, with their mean force shown (solid horizontal line). The bottom panel shows the half-life of the DNA tethers from the constant-force stretching experiments (with 60 pN tension). Data under each condition were obtained from ~140 biologically independent traces under constant-velocity stretching (dot plot; mean indicated as a solid horizontal line) and from *n* ≥ 3 biologically independent sample chambers, each with ≥30 traces under constant-force stretching (dot plot; also shown are mean ± s.e.m.). **b**, Cartoons illustrating the requirement for ATP hydrolysis in etoposide-enhanced topo II dynamics. Etoposide can stabilize both the cleavage complex and topo II capture of a DNA crossover only in the presence of ATP hydrolysis. In this model, whereas ATP hydrolysis promotes DNA-gate opening and T-strand transfer, etoposide stabilizes the DSB created by open DNA-gate and promotes the closure of the C-gate. As a result, topo II can stably capture both the G-segment and the T-segment. Source data

We found that, for topo II-mediated DNA loop capture, etoposide did not strengthen the loop rupture force without ATP and only slightly strengthened the loop rupture force with the non-hydrolyzable ATP analog AMP-PNP (Fig. 4a). Thus, topo II can still capture DNA loops without ATP, but hydrolyzable ATP is required for the tightened loop capture in the presence of etoposide. Because ATP hydrolysis is known to promote strand passage^32^, these findings suggest that etoposide leads to trapping of the T-segment between the DNA-gate and a closed C-gate after strand passage (Fig. 2c). For topo II-mediated DSBs, etoposide only slightly increased DNA breakage without ATP or with AMP-PNP (Fig. 4a). Thus, etoposide can significantly enhance topo II-mediated DSB formation in the presence of hydrolyzable ATP (Fig. 4b).

By comparison, we found that ICRF-187, a topo II catalytic inhibitor that stabilizes the dimer interface of the two ATPase domains near the N-gate^33^, only moderately increased the DNA loop rupture force and did not enhance the appearance of topo II-mediated DNA breaks (Fig. 4a). When etoposide was present together with ICRF-187, the DNA loop rupture force was similar to that with etoposide only, but the topo II-mediated DNA tether breakage force was higher under constant-velocity stretching, and the tether half-life was longer under constant-force stretching, compared with those of the etoposide-only condition. These findings are consistent with ICRF inhibitors reducing the ATP hydrolysis rate of topo II^34,35^, which may, in turn, reduce the frequency of etoposide-induced DSBs.

The ability of etoposide to promote the trapping of DNA loops and stabilize DSBs only in the presence of hydrolyzable ATP is notable (Fig. 4b). These observations provide evidence that a DNA-gate that has entered into a cleavage-competent state with a G-segment can be stabilized by etoposide to secure the DNA break; entry into this state also stabilizes closure of the C-gate, providing a mechanism to prevent subunit dissociation while the target DNA remains cleaved^4,36^. Our findings further indicate that etoposide-induced stabilization of the C-gate, when formed under normal ATP cycling conditions, can lead to a topological lock of both the G-segment and the T-segment.

### Etoposide converts topo II into a stronger roadblock

**Fig. 5:**
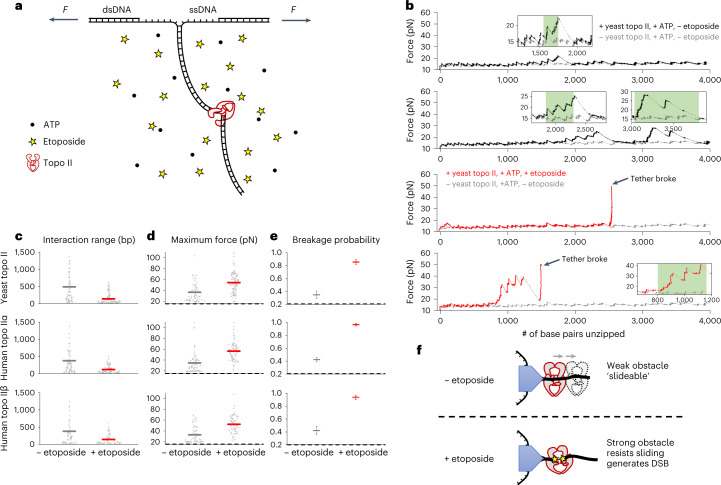
Etoposide reduces topo II mobility along DNA and enhances topo II resistance to removal. **a**, DNA unzipping configuration. DNA template was incubated with a low concentration of topo II (1 pM yeast topo II, 2 pM human topo IIα or 2 pM for human topo IIβ) and 1 mM ATP, with or without 100 µM etoposide. Bound topo II was detected via mechanical unzipping of the DNA. Most of the traces showing any bound protein should contain a single topo II (Extended Data Fig. 6b). **b**, Representative unzipping traces for DNA in the presence of yeast topo II. Inset panels show a zoomed-in view at the force clusters. Topo II sliding (green regions) is characterized by unzipping features similar to the underlying naked DNA baseline (gray curve) but with an elevated unzipping force. **c**, Topo II interaction range was calculated by summing all consecutive unzipping regions with force ≥2 pN above the DNA unzipping baseline for each trace. For each condition, data were pooled from *n* ≥ 120 biologically independent unzipping traces (dot plot), with the mean indicated (solid horizontal line). The same traces and statistics were used for the analysis of **d** and **e**. **d**, The maximum rupture force was determined from each bound trace. For each condition, data were pooled from all traces (dot plot) with the mean shown (solid horizontal line). The horizontal dashed lines (along the axes) indicate the unzipping force baseline for naked DNA alone (15 pN). **e**, The mean probability of tether breakage of the bound traces, defined as the fraction of unzipping tethers that broke without reaching at least 80% of the full length, was determined from all traces with bound proteins. Error bars are s.e.m. values calculated assuming a binomial distribution of the breakage probability. Horizontal dashed lines (along the axes) indicate the probability of tether breakage for naked DNA alone. **f**, Implications. Etoposide in the presence of ATP converts a bound topo II into a strong roadblock for removal by a motor protein, and its removal leads to a DSB. Source data

It has been suggested that a topo II molecule trapped on DNA by the action of a poison such as etoposide could become a roadblock to motor protein progression and disrupt DNA processing^37,38^. To better understand the physical nature of a topo II barrier, we measured the difficulty of removing a bound topo II by mechanically unzipping DNA through the bound enzyme using the DNA unzipping mapper technique^39,40^ (Fig. 5a and Extended Data Fig. 1b).

**Extended Data Fig. 6:**
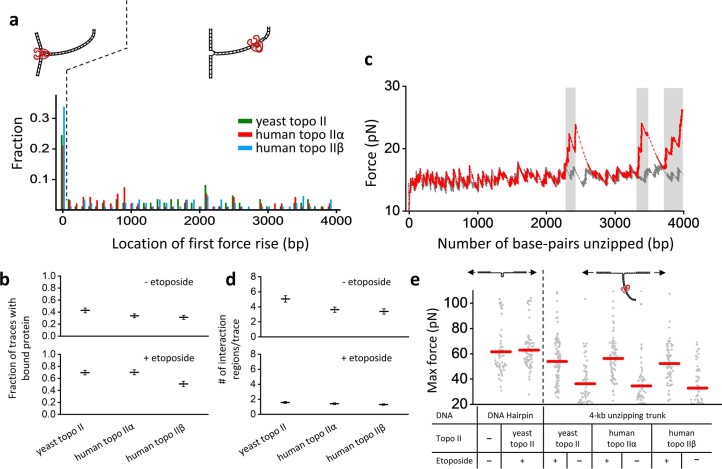
Unzipping signature of DNA with bound topo II (no etoposide) and control unzipping experiments to confirm DSB generation. (a) Location of the initial force rise of a bound topo II on the unzipping segment. Topo II had a higher binding preference to the unzipping fork junction but was bound somewhat randomly along the unzipping segment. Since the goal of the experiment was to investigate topo II sliding on a linear template, we did not consider the bound population at the fork (within the first 50 bp from the fork junction) in all subsequent analyses. (b) Fraction of traces with bound protein. Experiments were conducted with 1 pM yeast topo II, 2 pM human topo IIα or 2 pM human topo IIβ (see Fig. 5a caption). For each condition, the fraction bound was calculated from *N* ≥ 120 biologically independent traces. Error bars are s.e.m. values assuming a binomial distribution for the bound fraction. The same traces and statistics were also used for the analysis in (d). (c) A typical unzipping trace (red curve) with multiple interaction regions (gray shades areas) in the presence of 1 pM yeast topo II and 1 mM ATP without etoposide. (d) The number of interaction regions per unzipping trace. Error bars are s.e.m. values of the pooled data. (e) Maximum force. To exclude the possibility that the tether breakage observed in the DNA unzipping experiments with topo II and etoposide was due to breakage in the Y arms, we ligated a short DNA hairpin to the DNA Y-arms, creating an unzipping segment of near zero length, and unzipped this template in the absence or presence of topo II and etoposide. For each condition, the maximum force was pooled from *N* ≥ 90 biologically independent traces (dot plot) with the mean indicated as a solid horizontal line. Also shown to the right are six dot plots from Fig. 5c for comparison. Source data

We found that topo II bound to DNA at random locations along the DNA sequence (Extended Data Fig. 6a,b) and displayed diverse force features (Fig. 5b). Although some traces showed binding at a single region, other traces indicated binding at multiple regions (Fig. 5b and Extended Data Fig. 6c,d), possibly corresponding to the formation of a DNA loop or binding of more than one topo II molecule. Many traces clearly showed an extended interaction region, where the force was elevated from the underlying naked DNA baseline while still following the baseline profile (Fig. 5b, insets), suggesting that topo II can slide along the DNA under the influence of a progressing unzipping fork.

**Extended Data Fig. 7:**
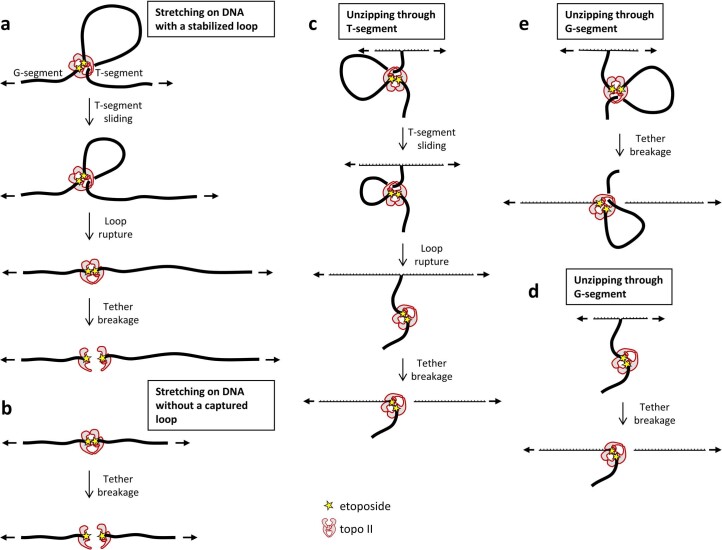
Topo II-mediated DNA loop, sliding, and tether breakage detected by either the DNA stretching or the DNA unzipping experiment. In a stretching experiment, if topo II traps a DNA loop (a), then the applied force on DNA slide topo II along the T-segment to reduce the DNA loop size, disrupt the DNA loop, and then separate the topo II dimer, leading to tether breakage. If topo II does not trap a DNA loop (b), then the applied force may directly separate the topo II dimer, leading to tether breakage. In an unzipping experiment, if the unzipping fork encounters the T-segment of a bound topo II that captures a DNA loop (c), the unzipping force may slide the topo II along the T-segment, disrupt the DNA loop, and then removes the 3’-end of DNA from the topo II, leading to tether breakage. In contrast, if the unzipping fork encounters the G-segment of a bound topo II (d and e), the unzipping force should always remove the 3’-end of DNA from the topo II, leading to tether breakage, regardless of whether the topo II captures a DNA loop.

When etoposide was present, topo II unzipping force signatures became more dramatic. A large fraction of bound traces (>80%) showed a sharp high-force rise before tether breakage, consistent with the unzipping fork encountering a tightly bound cleavage complex (Fig. 5b). Most of the remaining traces showed some sliding behavior where the force rose gradually over some distance, which was typically followed by tether breakage (Fig. 5b). This response is consistent for what would be expected if topo II were bound to two segments in a DNA loop and the unzipping fork encountered the T-segment first (Extended Data Fig. 7). In this case, unzipping might slide topo II along the T-segment until this strand is released, after which the cleavage complex is encountered. Control experiments confirmed that the tether breakage detected in the assay was not due to DNA end anchor detachment or topo II-mediated DNA breaks in the DNA adaptor arms (Extended Data Fig. 6e).

The interaction range, defined as the region of DNA with a force rise above the DNA baseline by at least 2 pN before tether breakage, reflects the sliding distance of topo II along DNA (Fig. 5c). In the absence of etoposide, the mean distance was 380–490 bp for the three topo II isoforms, of the same order of magnitude of the DNA loop size, suggesting that DNA loop formation likely contributes to topo II mobility on DNA. The presence of etoposide greatly reduced the mean distance to 120–140 bp. Therefore, etoposide restricts topo II’s ability to slide along DNA. The mean maximum force detected in the topoisomerase-bound traces (Fig. 5d), which served as a measure of the obstacle to unzipping, increased substantially from about 20 pN without etoposide to about 39 pN with etoposide, above the naked DNA baseline (15 pN). This indicates that etoposide converts a bound topo II into a much strong barrier for unwinding. In addition, etoposide increased the probability of a tether breakage from about 38% to about 92% (Fig. 5e). Thus, the presence of etoposide greatly increases both the resistance of topo II to removal and the susceptibility of the DNA tether to breakage.

Because the unzipping fork mimics motor progress through a bound protein, these results suggest that a motor protein can slide a bound topo II in the absence of etoposide (Fig. 5f). However, the presence of etoposide and ATP converts topo II into a strong roadblock that resists removal by a motor protein, and the eventual removal of topo II leads to a DSB.

### Etoposide induces prolonged pausing of topo II

**Fig. 6:**
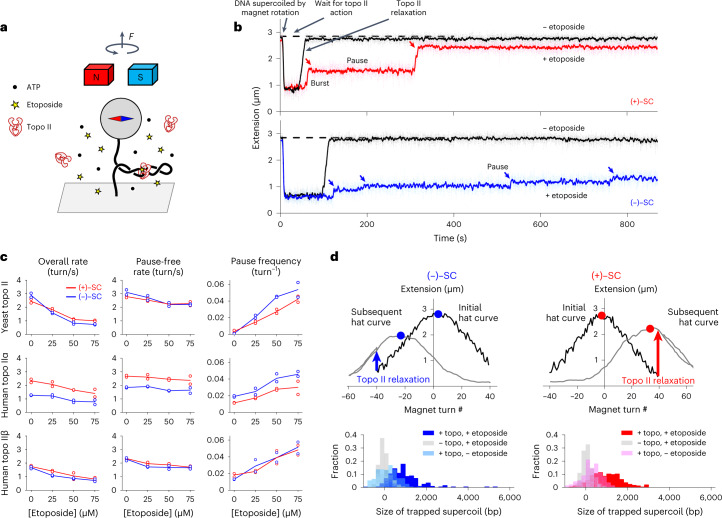
Etoposide induces topo II to ‘burst-and-pause’ during supercoiling relaxation. **a**, DNA was torsionally constrained between a magnetic bead and the surface of a coverslip and incubated with a low concentration of topo II (0.6 pM yeast topo II, 1 pM human topo IIα or 1 pM human topo IIβ) with 1 mM ATP and with or without etoposide. While held under a force of 0.22 pN, the DNA was supercoiled by rotation of a pair of magnets to buckle the DNA and form DNA plectonemes, allowing measurement of the extension versus magnetic turn relation (also referred to as a hat curve). **b**, Representative traces of supercoiling relaxation activity of yeast topo II with (black) or without (red and blue) 50 µM etoposide. Raw data were acquired at 10 Hz (dotted curves) and filtered using a sliding window of 25 points (solid curves). The horizontal dashed line indicates the expected extension of the relaxed 12.7-kb DNA tether under 0.2 pN. **c**, Mean relaxation rate, pause-free rate and pause frequency for topo II. At each etoposide concentration, data were pooled from *n* = 2 biologically independent sample chambers, each with ~90 traces, with the means connected by a solid line. **d**, Trapped DNA loop size. The top row shows example traces for determination of the size of the supercoiled loop trapped by yeast topo II. For each trace, after the initial hat curve, the magnets were rotated to −40 turns for (+) supercoiling experiments and +40 turns for (−) supercoiling experiments to measure topo II supercoiling relaxation, which increased the DNA extension. After 600 seconds, a second hat curve was obtained by rapid rotation of the magnets. A reduction in the hat curve height provides a measure of the size of the trapped supercoil. Two control experiments are also shown to provide uncertainties of the measurements. Data were pooled from ~180 biologically independent traces for each condition. SC, supercoil. Source data

Although etoposide is known to interfere with topoisomerase supercoiling relaxation, the dynamics of this interference have yet to be fully elucidated. To probe how etoposide interferes with topo II relaxation of DNA supercoils, we directly monitored topo II enzymatic activity using an MT supercoiling assay^20,41^ (Fig. 6a).

**Extended Data Fig. 8:**
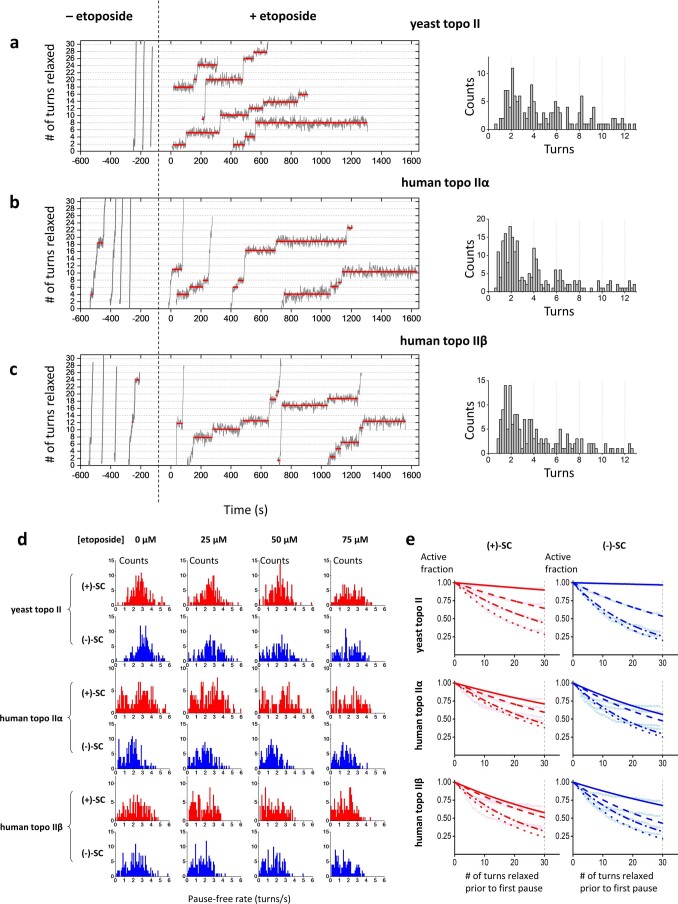
Detailed analysis of topo II’s ‘burst-and-pause’ on supercoiled DNA in the presence of etoposide. (a-c) Typical traces of the converted number of helical turns relaxed versus time for yeast topo II (a), human topo IIα (b), and human topo IIβ (c) acting on (-) supercoiled DNA under 0.2 pN in the presence of 1 mM ATP with or without etoposide. Data were smoothed with a 5-s sliding window filter. A pause-detection algorithm was used to detect pausing states (red). Shown on the right are histograms of the number of helical turns relaxed between adjacent pauses in the presence of etoposide of yeast topo II, human topo IIα, and human topo IIβ. A majority of the peaks occurred at integer multiples of 2 turns. (d) Histograms of the average pause-free velocity of supercoiling relaxation between consecutive pauses (one value for each trace) on both (+) and (-) DNA supercoils for yeast topo II, human topo IIα, and human topo IIβ in the presence of varying concentrations of etoposide. (e) For each relaxation trace, the number of turns relaxed by topo II prior to entering the first pause was measured, and these turn values were binned to create an active fraction plot. A single exponential function was used to fit the distribution to obtain the characteristic number of superhelical turns between pauses for varying etoposide concentrations. The inverse of this characteristic turn number was the pausing frequency of topo II. Shown are fits for conditions with no etoposide (solid), 25 µM etoposide (dashed), 50 µM etoposide (dashed-dotted), and 75 µM etoposide (dotted). Source data

In the absence of etoposide, the topo II isoforms relaxed DNA with minimal pausing (Fig. 6b and Extended Data Fig. 8a–c). However, in the presence of etoposide, processive DNA relaxation was abruptly interrupted by long pauses, giving rise to a ‘burst-and-pause’ behavior (Fig. 6b and Extended Data Fig. 8a–c). Detailed analysis of turns relaxed by topo II between pauses showed that pausing occurred predominantly at integer multiples of 2 turns (Extended Data Fig. 8a–c), a value that matches the expected change in the supercoiling state after each catalytic cycle^20,42^.

**Extended Data Fig. 9:**
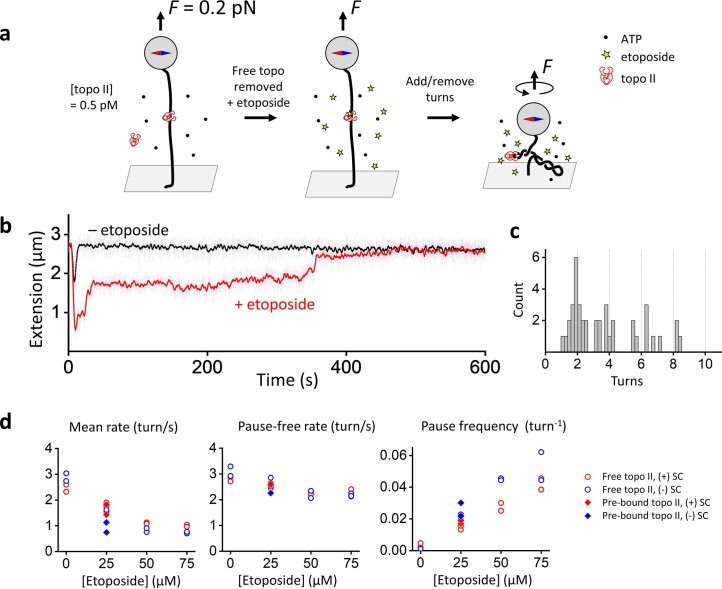
Burst-and-pause behaviors of yeast topo II in a pre-binding experiment. (a) To reduce the contribution of multi-topo during supercoiling relaxation, we performed a pre-binding experiment using a low concentration topo II. The DNA tether was held under 0.2 pN in a torsionally relaxed state, and 0.5 pM yeast topo II was introduced into the flow cell and incubated for 3 minutes (left). Subsequently, to remove unbound topo II, the flow cell was flushed with the topo reaction buffer with etoposide (middle). Then 40 turns were quickly added, and the tether extension was monitored to detect supercoil relaxation activity (right). The majority of the tethers (~ 80% within the first 10 s) showed no relaxation activity, which ensures single topo activity in the remaining traces with any supercoil relaxation activity. (b) Representative traces of supercoil relaxation activity of yeast topo II with or without 25 µM etoposide. Similar to the experiments in Fig. 6, yeast topo II quickly relaxed DNA supercoiling to completion without etoposide but experiences frequent pausing, interspersed by bursts of relaxation with etoposide. (c) Histogram of the number of turns relaxed between consecutive pauses with etoposide shows a similar pattern as shown in Fig. 6: etoposide induces topo II to pause after one-to-multiple catalytic cycles (1 cycle = 2 turns). (d) Overall relaxation rate, pause-free rate, and pause frequency of yeast topo II with 25 µM etoposide on (+) or (-) supercoiled DNA in the pre-binding experiment. For each condition, data were pooled from *N* = 2 biologically independent sample chambers (dot plot), each with ~ 35 traces for each chamber. For comparison, also shown are the corresponding data of Fig. 6c for conditions with free topo II, which show reasonable agreement with the results from the pre-binding experiment. Source data

Our data show that etoposide does not uniformly slow down actively relaxing topo II action during the process of topo II relaxation of supercoiled DNA. Instead, etoposide allows topo II to proceed for multiple enzymatic cycles with minimal interference but then sporadically jams topo II for a prolonged duration. For all three topo II isoforms, the overall rate of relaxation (including the pauses) decreased with an increase in etoposide concentration (Fig. 6c), and the extent of rate reduction is in reasonable agreement with that of a previous bulk study^32^. Intriguingly, the relaxation rate between pauses was essentially independent of etoposide concentration (Fig. 6c and Extended Data Fig. 8d). However, the pausing frequency was highly dependent on etoposide concentration and increased roughly linearly with an increase in etoposide concentration (Fig. 6c and Extended Data Fig. 8e). To ensure that these observations reflected the behavior of single topo II molecules, we incubated topo II at a very low concentration with the DNA substrate before removing any free topo II before measurements (Extended Data Fig. 9). Supercoiling relaxation assays under these conditions yielded a nearly identical relationship of the pausing frequency to etoposide concentration as shown in Fig. 6c.

The observed behaviors also reveal a supercoiling chirality dependence that is isoform specific to the topoisomerases being examined. Whereas human topo IIα shows a mean rate of relaxation about twice as fast on (+) supercoiled DNA than on (−), human topo IIβ and yeast topo II showed minimal chirality dependence (Fig. 6c). The chirality dependence of human topo IIα results from a combination of a faster pause-free relaxation rate and a lower pause frequency on (+) supercoiled DNA (Fig. 6c). In contrast, topo IIβ showed no detectable chirality dependence. Indeed, previous biochemical studies also found that human topo IIα prefers (+) supercoiled DNA, whereas human topo IIβ has no such preference^26,43,44^. The presence of etoposide did not substantially alter the chirality preference.

The observed prolonged pausing indicates that topo II was trapped in a stable state in the presence of etoposide. To determine if this state corresponds to a configuration of a stable topo II-mediated DNA loop, we measured DNA length under tension first before topo II introduction and then after topo II relaxation for 600 seconds (Fig. 6d). We found a pronounced reduction in the DNA extension after topo II relaxation in the presence of etoposide, both on (+) and (−) supercoiled DNA (Fig. 6d). This reduction is consistent with trapping supercoiled loops by topo II in the presence of etoposide. Control experiments without etoposide showed minimal detectable loop trapping (Fig. 6d). The loop size distributions are broad, with a mean of around 1,000 bp.

Thus, etoposide not only induces prolonged pausing of topo II supercoiling relaxation but also induces topo II to trap supercoiled DNA loops. Thus, the observed pausing is likely due to topo II’s inability to escape from a stably trapped DNA looping state. These findings also show that etoposide promotes the trapping of not only topologically relaxed DNA loops but also supercoiled DNA loops.

## Discussion

**Extended Data Fig. 10:**
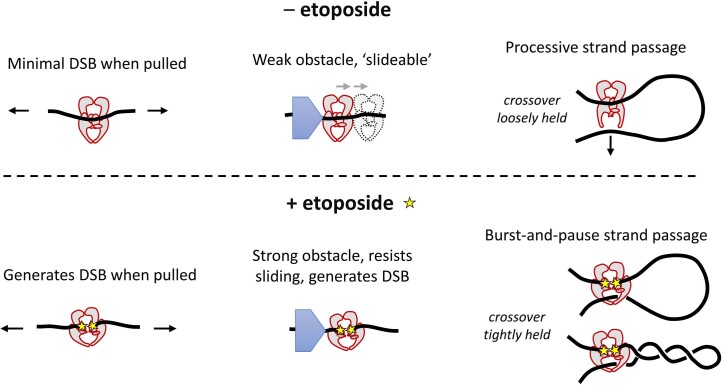
Dynamic consequences of etoposide. In the absence of etoposide, the DNA-topo II cleavage complex is short-lived, and a bound topo II is a weaker and mobile roadblock that does not create any significant DSBs. Because topo II quickly releases the T-strand after completing the strand passage, topo II does not stably hold onto a DNA crossover. This allows topo II to processively relax DNA supercoils. In the presence of etoposide and hydrolysable ATP, topo II stabilizes a DSB, forms a tight DNA loop, resists removal, and shows a “burst-and-pause” behavior during DNA supercoiling relaxation. The prolonged pausing is likely a result of topo II’s inability to escape from the stably trapped DNA looping state.

In the present work, we investigated the action of the topo II poison etoposide on the molecular mechanisms and dynamics of three topo II homologs (Extended Data Fig. 10) using three single-molecule mechanical manipulation techniques (DNA stretching, unzipping and twisting). We found that, even in the absence of etoposide, topo II can compact DNA by forming DNA loops (Figs. 1c and 2b,d,e), likely as part of its normal catalytic cycle. This compaction does not require ATP (Fig. 4a), suggesting that it is formed by the N-gate transiently capturing a T-segment, an interaction that was reported previously for yeast topo II^22^. The degree of DNA compaction is topo II isoform specific, with yeast topo II and human topo IIα being more effective than human topo IIβ at trapping a DNA loop. The C-terminal domain of human topo IIα has been suggested as a DNA binding element, but not human topo IIβ^26^, possibly facilitating DNA loop capture.

We observed that etoposide enhances topo II entrapment of DNA loops for all three isoforms only in the presence of hydrolyzable ATP (Fig. 4a), indicating that the drug stabilizes a state where the T-segment is likely trapped between the DNA-gate and a closed C-gate after strand passage upon ATP hydrolysis. Thus, the single-molecule data have captured a new and important consequence of etoposide binding that has hitherto been overlooked in the field. The degree of compaction is dependent on the concentrations of topo II and etoposide (Fig. 2 and Extended Data Fig. 4d). Given that the topo II concentrations used in this work (0.5–10 pM) are orders of magnitude smaller than those occurring in vivo (on the order of 1 μM)^45,46^, such compaction may have significant implications for how topo II restructures DNA in vivo. A topo II molecule bound to etoposide could lock two distant DNA segments from the same chromosome or from two different chromosomes, significantly altering chromosome structure and topology.

We directly reveal a DNA break of a topo II cleavage complex by mechanical separation of a topo II dimer interface (Figs. 2f, 3 and 4a). Our finding that etoposide enhances topo II-mediated DSB formation only in the presence of hydrolyzable ATP (Fig. 4a) supports the proposed asynchronization of ATP hydrolysis of the two bound nucleotides^32^, in which only one ATP is hydrolyzed to allow T-segment transport. When etoposide is present, we posit that the inability to religate the G-segment DNA after T-segment passage interferes with the hydrolysis of the second bound nucleotide, effectively jamming the enzyme.

Stably bound topo II has been proposed to interfere with transcription activity in vivo^38^. Etoposide could enhance the generation of a DSB by different types of mechanical forces that could either separate a topo II dimer or slide topo II along DNA (Extended Data Fig. 7). Our studies show that DNA-bound topo II is mobile in the absence of etoposide, but it resists relocation and removal in the presence of etoposide (Fig. 5). Topo II’s persistent interactions with DNA are likely facilitated by its binding to two segments of a loop, thereby reducing DNA dissociation. We found that etoposide greatly enhances the probability of forming a topo II-mediated DSB during DNA unzipping, but complete tether breakage (which corresponds to the full separation of the two DNA strands within the enzyme) requires significant force. This result suggests that, although topo II is covalently linked only to the 5′ strand of the cleaved DNA end, the enzyme still tightly clamps the 3′ strand, resisting DNA duplex separation and protein sliding by motor proteins.

Although it has been shown that etoposide significantly slows down topo II during multiple turnover strand passage events on catenated DNA substrates, such as kinetoplast DNA^32^, the molecular basis for this effect has not previously been fully understood. We found that etoposide interferes with the catalytic activity of topo II by introducing prolonged pausing during processive relaxation of supercoiled DNA in a ‘burst-and-pause’ fashion and that the effect of the drug can be more pronounced for some enzyme isoforms depending on the chirality of the DNA substrate (Fig. 6c). We also found that topo II traps supercoiled DNA loops in the presence of etoposide (Fig. 6d), suggesting that the paused state might be due to the inability of topo II to escape from the loop-trapped state during DNA supercoiling relaxation.

The work described here provides a detailed dissection of the molecular mechanisms by which the topo II poison etoposide corrupts the action of topo II on supercoiled and non-supercoiled DNA templates. It reveals that dynamic interactions of yeast topo II, human topo IIα and human topo IIβ with DNA are remarkably similar, all having a similar ability to resist dimer separation under DNA stretching and to resist sliding along DNA under DNA unzipping. However, they show significant differences in DNA compaction and supercoiling relaxation chirality. These complex behaviors are coming to light via highly sensitive single-molecule assays. We anticipate that the techniques used here will be beneficial in the study of a broad range of topo catalytic inhibitors and poisons, serving as sensitive screening tools that can provide insights into defining drug mechanisms and enzyme isoform specificity.

## Methods

### Protein purification and DNA template construction

Expression and purification of yeast topo II, human topo IIα and human topo IIβ were the same as previously described^47^. In brief, cultures were grown in selection media; protein expression was induced; cells were harvested by centrifugation; and resulting pellets were flash-frozen. Pellets were lysed, and lysate was loaded onto a series of purification selection columns, resulting in a purified protein free of tags. The protein purity was assessed via SDS–PAGE, and selected fractions were filter concentrated and flash-frozen for storage.

The torsionally constrained DNA construct used for stretching and twisting experiments contained a 12,688-bp DNA segment (~50% GC content), flanked by a ~500-bp multi-labeled tethering adaptor at each end (Extended Data Fig. 1a). The 12.7-kb DNA was amplified from lambda DNA (New England Biolabs (NEB), N3011S) using the primers CTACCCGAGTGCGTGACAT and CCAGTCTCGTGAAGCGGTA with Phusion DNA polymerase (NEB, M0530S). The construct was then purified via PureLink spin-column purification kit (Invitrogen, K310002) and subsequently cleaved with AvaI (NEB, R0152S) and BssSI-v2 (NEB, R0680S) to produce unique DNA overhangs. The final DNA construct was obtained by ligating ~500-bp multi-labeled adaptors to each end of the end-cleaved 12.7-kb DNA with T4 Ligase (NEB, M0202S). These multi-labeled adaptors were amplified from the plasmid pNFRTC (or pMDW111) and labeled via polymerase chain reaction (PCR) with either 24% of dATP replaced by biotin-14-dATP (Invitrogen, 19524-016) or 24% of dTTP replaced by digoxigenin-11-dUTP (Roche, 11093088910). The DNA primers for the biotin-labeled adaptor are CAGTCACGAGGTTGTAAAACG and ACGCCAAGCTTCCACATC, and the DNA primers for the digoxygenin-labeled adaptor are GGGTAACGCTCGGGTTTTCC and ACGCCAAGCTTCCACATC. To create the DNA overhangs, the resulting PCR products were digested with BssSI-v2 (NEB, R0680S) for the biotin adaptor or AvaI (NEB, R0152S) for the digoxygenin adaptor. Ligation products were gel purified to obtain a homogenous DNA sample.

The unzipping experiments used a 4-kb unzipping DNA segment (~50% GC content) ligated to a pair of DNA Y-arms^40^ with a total length of 2 kb (Extended Data Fig. 1b). The 4-kb DNA unzipping trunk was amplified from lambda DNA (NEB, N3011S) using the DNA primers CCCGCAGCTACTGGATTAAACAAGCC and GTAGCACCAAAGGAAACCATCACCCA with Phusion DNA polymerase (NEB, M0530S). The PCR product was digested with AlwNI (NEB, R0514S), gel purified and ligated to the 2-kb DNA Y-arms^40^ using T4 Ligase (NEB, M0202S).

### Single-molecule sample chamber preparation

The hydrophobic nitrocellulose-coated grease microfluidic sample chamber was prepared following our previous report^41^. Before the experiment, a sample chamber was incubated with 20 ng µl^−1^ of anti-digoxygenin (Roche, 11333089001) for 30 minutes at room temperature. For MT experiments, fiducial marker beads (Dynabeads MyOne Streptavidin T1, 65601) coated with 500-bp biotin and digoxygenin-labeled DNA^41^ were bound to the surface of the chamber. The surface was then passivated by flushing the chamber with 25 mg ml^−1^ of β-casein (Sigma-Aldrich, C6905) and incubating for 1 hour at room temperature.

For OT DNA stretching experiments and MT DNA twisting experiments, 1.5 pM of the 12.7-kb DNA in DNA dilution buffer (10 mM Tris-Cl pH 7.8, 50 mM NaCl, 1 mM EDTA and 1.5 mg ml^−1^ of β-casein) was incubated in the sample chamber for 15 minutes at room temperature. Streptavidin-coated 500-nm polystyrene beads (OT experiments) or 1-μm superparamagnetic beads (Dynabeads MyOne Streptavidin T1, 65601; MT experiments) were then introduced into the chamber. For the OT unzipping experiments, 6 pM of the DNA Y-arms ligated to the unzipping trunk template was incubated in the sample chamber, followed by an incubation of the streptavidin-coated 500-nm polystyrene beads.

Unless stated otherwise, topo II was diluted to 10× the working concentration right before the experiment using topo dilution buffer (30 mM Tris pH 7.8, 500 mM KCl, 10% glycerol (v/v), 0.5 mM TCEP and 0.1 mg ml^−1^ of β-casein). All experiments were carried out in topo reaction buffer (10 mM Tris-HCl pH 7.8, 50 mM NaCl, 50 mM KCl, 3 mM MgCl_2_, 0.1 mM EDTA, 1 mM DTT, 0.5 mM TCEP, 1 mM ATP (Roche, 11140965001) and 1.5 mg ml^−1^ of β-casein) in a soundproof room at a temperature of 23 °C. For the stretching experiments with no ATP, we modified the buffer to contain 2 mM MgCl_2_ to maintain a similar free MgCl_2_ concentration as in the topo reaction buffer.

### DNA stretching assay

DNA stretching experiments used a single-beam OT setup^17^ under one of two modes: constant velocity or constant force. In a sample chamber with anchored 12.7-kb DNA tethers, topo II was diluted to the desired concentration in the topo reaction buffer with or without 100 µM etoposide, introduced into the sample chamber and incubated for 10 minutes at room temperature. Subsequently, the sample chamber was sealed with high vacuum grease (Dow Corning, 1597419). Prior bulk biochemical studies show that etoposide binding has an equilibrium-binding constant of ~5 μM^11,48^. Thus, 100 μM etoposide should allow a near-saturating occupancy condition.

On the OT setup, for each tether, the bead was trapped, and the tether’s anchoring position was determined by stretching the tether up to 0.5 pN along both positive and negative directions, first along the *x* axis and then along the *y* axis. These low-force data were used to determine the degree of DNA compaction. Subsequently, the tether was allowed to relax before being stretched to high forces by moving the coverslip along the *x* axis. In the constant-velocity mode, the coverslip was moved at a fixed speed of 200 nm s^−1^. In the constant-force mode, the laser power was fixed, and the coverslip was moved at 8,000 nm s^−1^ to abruptly increase the tension to 60 pN. Subsequently, the coverslip’s position was modulated to maintain a constant force of 60 pN on the DNA.

In the absence of topo II, a fraction of tethers broke below or at the DNA overstretch transition (~65 pN), indicating that they were likely not torsionally constrained to surfaces. The remaining tethers reached a much higher force without displaying any force features of the overstretch transition, consistent with being torsionally constrained. Some tethers did not break before escaping from the optical trap after reaching the maximum trapping force (about 110 pN at the laser power used). If this occurred, the tether breakage force was indicated by the escape force, which provides a conservative estimate of the breakage force for that trace.

For stretching experiments with etoposide (Sigma-Aldrich, E1383) and AMP-PNP (Roche, 10102547001), we modified the above procedure because AMP-PNP can prevent topo II binding to DNA without free ends^49^. We diluted topo II in an ATP-free buffer (10 mM Tris-HCl pH 7.8, 50 mM NaCl, 50 mM KCl, 2 mM MgCl_2_, 0.1 mM EDTA, 1 mM DTT, 0.5 mM TCEP and 1.5 mg ml^−1^ of β-casein), introduced it into the sample chamber and incubated at room temperature for 30 minutes to allow equilibration of topo II binding to DNA. We then flushed the chamber with a buffer that contained AMP-PNP and etoposide (10 mM Tris-HCl pH 7.8, 50 mM NaCl, 50 mM KCl, 3 mM MgCl_2_, 0.1 mM EDTA, 1 mM DTT, 0.5 mM TCEP, 1 mM AMP-PNP, 1.5 mg ml^−1^ of β-casein and 100 μM etoposide) and performed the stretching experiments as described above.

To generate DNA tethers with different numbers of nicks, the surface-anchored DNA was incubated with various Nt.BsmAI (NEB, R0121S) concentrations (0.1, 1, 2, 5 and 30 U ml^−1^) diluted in the nicking buffer (10 mM Tris-Cl pH 7.8, 10 mM NaCl, 0.5 mM MgCl_2_, 0.5 mM TCEP and 1.5 mg ml^−1^ of β-casein). After 15 minutes of nicking, the nickase was removed by flushing the flow chamber with DNA dilution buffer, and the topo reaction buffer was introduced for the stretching experiment. A constant-velocity or constant-force experiment was conducted as described above.

Data were acquired at 10 kHz and decimated by averaging to 1 kHz. Raw data were converted into force and DNA extension as previously described^50^. For DNA loop disruption peak detection, the data were low-pass filtered with a sliding window of 25 ms.

The OT measurements yielded force versus time *F*(*t*) and the corresponding extension versus time *x*(*t*). To convert these data to DNA contour length *L*_*o*_(*t*), we used the modified Marko–Siggia model^19^, which states that the force on the DNA depends on the normalized extension $F\left( {\frac{x}{{L_o}}} \right)$, as long as the persistence length and stretch modulus are determined. Therefore, once force *F*(*t*) and extension *x*(*t*) are measured for any timepoint *t*, the corresponding DNA contour length *L*_*o*_(*t*) is also determined. The DNA contour length *L*_*o*_(*t*) can then be converted to the number of base pairs (0.338 nm per bp). This strategy was initially developed to determine RNA polymerase position on the DNA during transcription stalling under an external force^51^ and later used to determine the DNA release from a nucleosome during mechanical disruption^17^.

### DNA unzipping assay

The DNA unzipping experiments used an OT setup. After the DNA unzipping template was tethered to the surface in a sample chamber, topo II was introduced and incubated for 10 minutes at room temperature. Subsequently, the chamber was sealed with high vacuum grease. For each DNA tether, the tether was stretched and subsequently unzipped by moving the coverslip along the *x* axis under a constant velocity of 400 nm s^−1^. For the control experiment with a DNA hairpin, a hairpin with the sequence 5′-/Phos/GCTATTTTTTTAGCTAG was ligated directly to the Y-arms before tethering the DNA to the surface. Subsequent unzipping experiments with the DNA hairpin template were done in the same way as for the 4-kb DNA unzipping trunk template.

Data were acquired at 10 kHz and decimated by averaging to 1 kHz. Raw data were converted into force, DNA extension and the number of base pairs unzipped as previously described^50^. For sequence alignment, the number of base pairs unzipped data was low-pass filtered with a sliding window of 25 ms.

To convert the measured force and extension data, we used a strategy similar to that described above for dsDNA, except that we simultaneously considered the elastic properties of both ssDNA and dsDNA under tension^52,53^.

### DNA twisting assay

The DNA twisting experiments used a custom-built MT setup^41^. The magnetic field was generated with two 0.25-inch cube neodymium magnets (K&J Magnetics, B444) arranged with their dipoles oriented in opposing directions, parallel to the optical axis of the microscope with a separation gap of 0.5 mm. Magnetic bead images were collected by a Nikon objective lens (Plan Apo ×20, 0.75 NA) on a 2.3-megapixel camera (Basler, acA1920-155um) at a frame rate of 10 Hz and an exposure time of 0.15 ms. The bead positions were tracked in three dimensions using an algorithm implemented in LabVIEW based on Omar Saleh’s source code^54^.

For the DNA twisting experiments, each multi-tagged 12.7-kb DNA molecule was torsionally anchored between a coverslip and a magnetic bead and held at 0.22 ± 0.03 pN (mean ± s.d.). This low force value was chosen to minimize the potential impact of the applied force on topo II speed^44^. Initially, the magnet was rotated around the zero-turns state to establish the ‘initial hat curve’ for each tether. Then, topo II was introduced into the sample chamber. Then, DNA was (+) or (−) supercoiled by rotating the magnets for 40 turns, and, subsequently, data were recorded for 900 seconds. Subsequent relaxation by topo II was reflected as an increase in the DNA extension. Afterwards, unbound topo II and ATP were removed by topo flushing buffer (10 mM Tris-HCl pH 7.8, 50 mM NaCl, 50 mM KCl, 2 mM MgCl_2_, 0.1 mM EDTA, 1 mM DTT, 0.5 mM TCEP and 1.5 mg ml^−1^ of β-casein). A ‘final hat curve’ for each tether was acquired for comparison as for the ‘initial hat curve’. Finally, the tether was wound to the surface to obtain a height offset for absolute length measurement. For the supercoil loop-trapping experiment, after 600 seconds of topo II relaxation, we quickly rotated the magnets at 10 turns per second around the current magnet position to capture a quick-winding hat curve of the tether to check for tether height reduction.

For the pre-binding experiments (Extended Data Fig. 9), after the ‘initial hat curve’ of the 12.7-kb DNA molecule was characterized and the tether was relaxed to a torsional free state under 0.2 pN tension, 0.5 pM yeast topo II was introduced into the sample chamber and incubated for 3 minutes. Then, the chamber was flushed twice with a copious amount of topo reaction buffer (10 chamber volumes for each flush), and −40 turns for (−) supercoiling experiments and +40 turns for (+) supercoiling experiments were quickly added, and the tether’s extension was recorded for 900 seconds. Afterwards, topo activity was stopped by topo flushing buffer; a ‘final hat curve’ was recorded; and the tether was finally wound to the surface.

### Computer simulation of DNA looping

The DNA loop size distribution from topo II binding reflects the probability of juxtaposing non-adjacent DNA regions along the same DNA molecule under no tension to form a DNA loop. We calculated looping probability, or the Jacobson–Stockmayer *J*-factor for DNA looping, assuming the DNA ends are at a 2-nm end-to-end distance in close contact and that they are free to adopt any orientation relative to each other. We considered two scenarios: a single topo II captures a naturally occurring DNA crossing via the formation of a loop, or a bound topo II bends the DNA significantly and facilitates the formation of a small loop whose ends are captured by a second topo II.

For the first scenario, the *J*-factor was obtained using a semi-analytical approximation from Douarche and Cocco^55^ with a 2-nm end-to-end distance and a 45-nm persistence length. For the second scenario, we constructed a coarse-grained model (Extended Data Fig. 3) of topo-bound DNA and used computer simulation to obtain the looping probability^24^. Looping of <200-bp dsDNA is energetically unfavorable due to the inherent DNA bending stiffness. The sharp DNA bend introduced by a bound topo II facilitates the formation of small DNA loops as it lowers the energy barrier of DNA bending. To calculate the looping probability of topo-bound DNA, we computed the *J*-factor of looping at the end-to-end distance of 2 nm and bending angle *α* as a function of total DNA loop size. In this approach, a biased potential $U = \frac{1}{2}k\left( {r - r_0} \right)^2$ is added to the total bending energy of the DNA chain to increase the statistics of looping events for the small end-to-end distance *r* near *r*_0_. The spring constant *k* was chosen as $\frac{{1.682}}{{25}} \times \exp ( - 0.008664 \ast L) \times (55 - 0.2 \ast \alpha )$ (pN.nm/bp^2^) where *L* is the total DNA contour length in base pairs and *α* is the bending angle in degrees. A pool of DNA trajectories is generated and equilibrated at room temperature using a standard MC procedure^24^. For each simulation, 10^5^ initial MC steps were used to equilibrate the system but then discarded, and the data were collected from the subsequent 1.5 × 10^6^ MC steps. A typical MC step involves a pivot rotation of the subchain within the original chain around a random axis that passes through a random vertex of the non-bent DNA with an angle randomly distributed in [−50°,50°]. After obtaining a set of the probability density function of the end-to-end distance, $P_{biased}(r)$, for different *U*(*r*), the unbiased $P^0\left( r \right)$ can be obtained using the weighted histogram analysis method^24,56^. The *J*-factor is then computed as $J\left( r \right) = P^0\left( r \right)/(4\pi r^2)$ and converted into nanomolar units using the Avogadro constant.

### Statistical analysis

All data were obtained from at least two sample chambers. Statistical details, including the number of traces and s.d. or s.e.m. values, can be found in the manuscript text, Methods subsections, figure captions and Source Data file. Additional details of data analysis are described below.

#### DNA loop disruption peak detection

For filtered stretching data, the disruption force peaks were identified using a MATLAB code called ‘peakfinder.m’, which can be found at https://www.mathworks.com/matlabcentral/fileexchange/25500-peakfinder-x0-sel-thresh-extrema-includeendpoints-interpolate. Only force peaks above 1 pN were selected. To avoid the irrelevant contribution from DNA overstretching, we set an upper threshold for the peak force at 60 pN.

#### DNA alignment algorithm

To improve precision and accuracy of unzipping data, we used custom software written in MATLAB^40^ to perform a cross-correlation optimization to align each ‘experimental force versus the number of base pairs unzipped’ curve with the corresponding theoretical curve using regions immediately preceding and after the protein disruption^57^.

#### Pause detection algorithm

To detect pauses in supercoiling relaxation by topo II, we employed an improved pause detection algorithm based on dwell-time analysis^40,58^. The extension data were low-pass filtered using a non-linear 2nd-order Savitzky–Golay filter with a time constant of 30 seconds. A multi-piece fit to the ‘initial hat curve’ converted extension to the turn state from which the number of helical turns relaxed over time was deduced. The number of turns relaxed was binned to 0.2-turn intervals, and the dwell-time at each bin was computed. Subsequently, clusters of adjacent bins with notable dwell-time (>2 seconds per bin) were identified, and each cluster was assigned as a single pausing state. The pausing regions were mapped back to the time domain by assigning data points within 0.4 turns around the identified pausing levels. Finally, the pausing turn state and pause duration at each state were refined by averaging all data points that belong to the same state.

The pause-free rate was calculated for the ‘bursting’ region before the first pause. After identifying the activity burst before the first pause, the raw data were filtered by a sliding window with a time constant of 5 seconds, and the instantaneous rate was obtained by performing a linear fit to the number of helical turns relaxed over time. The pause-free rate during the first bursting event of each trace was obtained by binning the instantaneous rate into the 0.5-turn bin and taking the median of the binned data.

Pause frequency was measured from the number of helical turns relaxed before entering the first pause. For traces without detected pause, the maximum number of turns relaxed before entering the pre-buckling region of the 12.7-kb DNA twisting under 0.22 pN (~30 turns) was assigned. A cumulative histogram of the relaxed turn numbers was constructed and fitted using a single exponential function to obtain the pause frequency.

### Reporting summary

Further information on research design is available in the Nature Portfolio Reporting Summary linked to this article.

## Online content

Any methods, additional references, Nature Portfolio reporting summaries, source data, extended data, supplementary information, acknowledgements, peer review information; details of author contributions and competing interests; and statements of data and code availability are available at 10.1038/s41589-022-01235-9.

## Supplementary information

Reporting Summary

## Data Availability

Relevant source data for the main figures and Extended Data figures are provided in the Source Data files. All other data that support the findings of this study are available from the corresponding author upon reasonable request. Source data are provided with this paper.

## Source data

Source Data Fig. 1 Statistical source data
Source Data Fig. 2 Statistical source data
Source Data Fig. 3 Statistical source data
Source Data Fig. 4 Statistical source data
Source Data Fig. 5 Statistical source data
Source Data Fig. 6 Statistical source data
Source Data Extended Data Fig. 1 Unprocessed gels
Source Data Extended Data Fig. 2 Statistical source data
Source Data Extended Data Fig. 3 Statistical source data
Source Data Extended Data Fig. 4 Statistical source data
Source Data Extended Data Fig. 5 Statistical source data
Source Data Extended Data Fig. 6 Statistical source data
Source Data Extended Data Fig. 8 Statistical source data
Source Data Extended Data Fig. 9 Statistical source data

## Code availability

Custom MATLAB code files are available on GitHub: https://github.com/WangLabCornell/NCHEMB-A220414677B.
