# Supplementary figures and images for: Etoposide promotes DNA loop trapping and barrier formation by topoisomerase II
Source: Nat Chem Biol. 2023 Jan 30;19(5):641–50. doi: 10.1038/s41589-022-01235-9 (PMC10154222; doi:10.1038/s41589-022-01235-9)

Yeast topo II

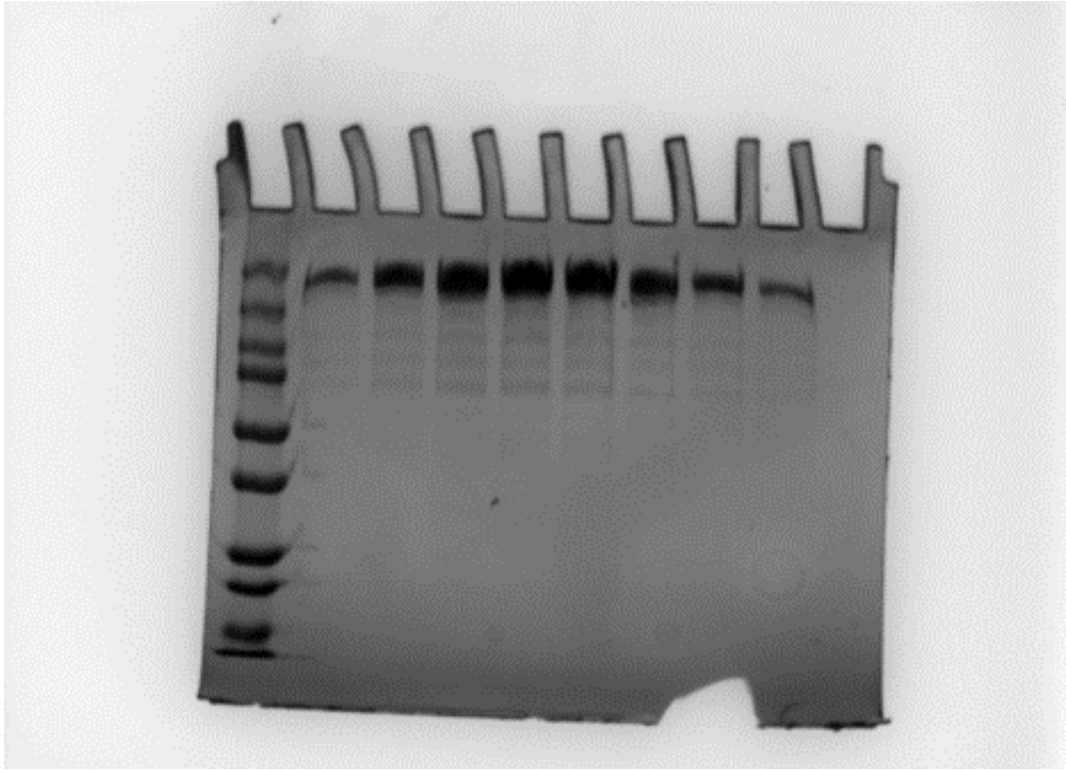

Human topo II $\alpha$  (left) and Human topo II $\beta$  (right)

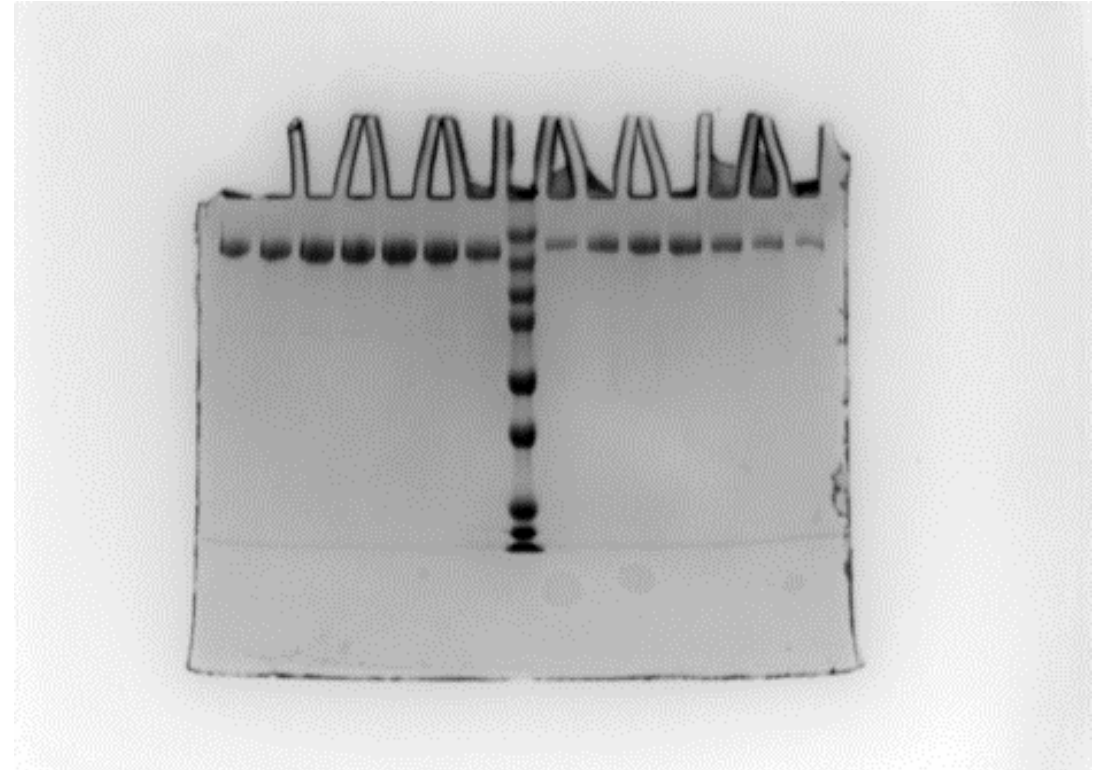

Supplement: Source Data Extended Data Fig. 1 — Unprocessed gels [file 41589_2022_1235_MOESM8_ESM.pdf]
